# Supplementary material for: Patterns of Intron Gain and Loss in Fungi
Source: PLoS Biol. 2004 Nov 30;2(12):e422. doi: 10.1371/journal.pbio.0020422 (PMC532390; doi:10.1371/journal.pbio.0020422)
Supplement: Table S1 — Also available at http://genes.mit.edu/NielsenEtAl/. (4.3 MB ZIP). [file pbio.0020422.st001.zip › NielsenEtAl/html/615.html]

AN1634.1.NCU02803.1.MG01583.1.FG06849.1


```
 CLUSTAL W (1.82) Multiple Sequence Alignments - Introns Inserted


Sequence 1: AN1634.1	767 aa
Sequence 2: FG06849.1	721 aa
Sequence 3: NCU02803.1	728 aa
Sequence 4: MG01583.1	674 aa
Alignment Length: 814 aa
Number Identitical Residues: 278 aa
Alignment Score (without introns) 14787


MG01583.1 	------------------------------------------------------------
NCU02803.1	------------------------------------------------------------
FG06849.1 	------------------------------------------------------------
AN1634.1  	MESQSILPPPPPPEDASAPPPPPEESAAPPPPPDVSAPPPPPEDLPPPPPEPEVKKKKVG
          	 .:.:  ........:::.......:::...... ::....... ........ .... .

MG01583.1 	-------MAPLDLEEILKKHKAAKAEAAK~PRFIPRGQRKKMEEEKKLKVEEEQKRQQEE
NCU02803.1	-MSTTSRREPPDLAALLRKKKEEEAAAAK~PRFIPKKERERLEAEKKAKEEEERKRKEEA
FG06849.1 	--------MPLDIEELLRKKKAADTAAAK~PRFIPKAERERLAAEKAKKEEDDKKRKASE
AN1634.1  	WGTKRPATTPLSVEELVRKKREADAAAAR0PKFMSKAERERLALEKRAKEVDAQRRRTNG
          	  :. .   * .:  :::*::  .: **: *:*:.: :*:::  **  *  : ::*: . 

MG01583.1 	LQKTRSELQKAREELAGLQRVRRQEQSRSRESDHSVPTGPRAMRSYDDDSFNGNGNGSN-
NCU02803.1	KPQPNGTNHNG-------NRMDGIQSHHNHNPQRNIPTGPKAMRYDDDRGPNGMSNGRDY
FG06849.1 	EEQKRKEEEQK-------WRSNGSSRPNESNGSGRVPTGPRSMNDGRDDRERDRDRDQ--
AN1634.1  	TPNGVDGMDLD-------TPSRGFRTPNGDSRS--IPTGPRAMRNGDGPTPTGPAAMR--
          	  :     .                  .  . .  :****::*.   .    .       

MG01583.1 	SNRNNNNNQRDRQDASTKTNGSRTAE------EVQEEKKYLERYTGPPAKVST-FSANKK
NCU02803.1	RDNRDNRDNRDRNQRGAKRGAPNDDEEKRAKMERNDEAELRARYMGPVVNQST-FSAKKK
FG06849.1 	--GRGRVRGKDRKGDKQGVGADKQSA------EDIEATLLRSRYLGPQVNQQSNFSAKKK
AN1634.1  	--SRNDS-GTTKSDKKVDKRFNEEDE------AAAQAALIKQRYMG--ADQTSNFSAKKK
          	   ..      :.         .            :      ** *  ..  :.***:**

MG01583.1 	RRRTTDQKFNFDWDPKDDTSQPWRYEETGAHERS--------------ANGATEQVRRKK
NCU02803.1	RRRTAANKFNFDWDADDDTSRPFDPIYAERQEP-------LVRLGG--YEMTEEMVMRKA
FG06849.1 	RMRTTEKKFNFEWDADEDTSRDNDPLYDRQTAVSHNG--SFAGIGGEFDDGAEERARKRA
AN1634.1  	RKRTTDRKFNFEWNAEEDTSGDYNPLYQHRHEANFFGRGRLAGFG---DDVADSVAKKYA
          	* **: .****:*:..:***             .  . .     .    : : . . :  

MG01583.1 	ATRDYNDPRLVP-----------------------------WQDKELSQMTTRDWRLFKV
NCU02803.1	EAIRRGDPETGEERARQYLEQHRRIKEME----QRKNLGKHWSEKKLEDMKERDWRIFKE
FG06849.1 	KMIAQRDPENGKERAEGIMEDFFRARDKARQRADRRGLGKHWSEKSLDDMRERDWRIFKE
AN1634.1  	KALEDRDREAGSIRAREILEMERRRREES----TRNQLDKHWSEKKLEHMRERDWRIFKE
          	      * .     :    .      .           .. *.:*.*..*  ****:** 

MG01583.1 	NLEIVTKGNNIPNPMRFWEESNLPHVLKDTIKQVGYTEPTPVQRAAIPIALQCRDLIGIS
NCU02803.1	NFGIATKGGAIPNPMRSWEESTLPRRLLDIVKNVGYDEPTPIQRAAIPIALQARDLIGVA
FG06849.1 	DFGIATKGGMIPNPMRSWQESNLPQRLLNIVDDVGYKDPSPIQRAAIPIALQARDLIGVA
AN1634.1  	DFNIATKGGSVPNPMRSWDESGLPKRLLELVDRVGYKEPTPIQRAAIPIAMQSRDLIGVA
          	:: *.***. :***** *:** **: * : :. *** :*:*:********:*.*****::

MG01583.1 	KTGSGKTAAFVLPMLSYIEP~LPPLNEVT--KTEGPYALILAPTRELATQIQAEVIKFAT
NCU02803.1	VTGSGKTAAFLLPLLVYISE~LPPLTEYN--KNDGPYALILAPTRELVQQIESEAKKFAT
FG06849.1 	VTGSGKTAAFLLPLLVYISD~LPPLDEIN--KHDGPYALIMAPTRELVQQIETEARKFAG
AN1634.1  	VTG---------------SE1LPRIDEFEWRKNDGPYAIVLAPTRELAQQIEIEAKKFTE
          	 **               .  ** : *    * :****:::******. **: *. **: 

MG01583.1 	RMGFTVVCLIGNKRTIEEDAFALRNGAEIIVATPGRLVDCLERHLLVLSQCSYVVLDEAD
NCU02803.1	PLGFTVVSIVGG-HSLEEQAFALRNGAEIIVATPGRLVDCLERRLLVFSQCCYTIMDEAD
FG06849.1 	PLGFRVVSIVGG-HQIEEQAYNLRDGAEIVVATPGRLLDCIERRLLVLSQCCYVIMDEAD
AN1634.1  	PLGFNVVSIVGG-HSFEEQAYSLRNGAEIIIATPGRLVDCIERRMLVLSQCCYVIMDEAD
          	 :** **.::*. : :**:*: **:****::******:**:**::**:***.*.::****

MG01583.1 	RMVDGGFEDSIHKILAALPPSNGKPDDRDAEDPNIMSKFLTPNLRYRQTVMYSATMPPSV
NCU02803.1	RMIDQGFEEPLTKILDAMPVTNEKPDTDDAENPQLMSRYVDGKDRYRQTMMYTATMPPIV
FG06849.1 	RMIDLGFEESVNKILDALPVTNEKPDTDEAENAQIMQRYLGGRDRYRQTMMYTATMPPLV
AN1634.1  	RMIDLGFEEPVNKILDALPVTNEKPDTEEAEDSSAMSRHLGSKDRYRQTMMYTATMPTAV
          	**:* ***:.: *** *:* :* ***  :**:.. *.:.:  . *****:**:****. *

MG01583.1 	ERIAKNYLKHPAMVTIGTIGEAVDTVEQQAMWVVSEDERRNKLRAMLNTYGTGKLVIVFV
NCU02803.1	ERIAKKYLRRPAIVTIGNAGEAVDTVEQRVEFVSGEDKRKKRLQEILNSGQFKPPIIVFV
FG06849.1 	ERIAKKYLRRPAIVTIGNAGEAVDTVEQRVEFVSGEDRRKKRLQEILSSGNFGPPIIVFV
AN1634.1  	ERIARKYLRRPAIVTIGSAGEAVDTVEQRVEMIAGEDKRKKRLGEILSSGDFRPPIIVFV
          	****::**::**:****. *********:.  : .**.*:::*  :*.:      :****

MG01583.1 	NTKSNCDAVAKDLKSSSFSAVTLHGNKTQDQREAALQSFRDGRTNVLVATDVAARGLDIP
NCU02803.1	NIKRNCDMVARDIKGMGYSAVTLHGSKTQEQREAALASLRNGQTDILVATDLAGRGIDVP
FG06849.1 	NIKRNCDAVARDIKQMGWSAVTLHGSKTQEQREAALGSVRAGHTQVLVATDLAGRGIDVP
AN1634.1  	NIKRNCDAIAREIKQWGFSSVTLHGSKTQEQREAALASVRNGSTDVLVATDLAGRGIDVP
          	* * *** :*:::*  .:*:*****.***:****** *.* * *::*****:*.**:*:*

MG01583.1 	DVSLVINFNMAGTIEVYTHRIGRTGRAGKEGMAITFCGPEDHGVL~YHLKQIMSKSQMSK
NCU02803.1	DVSLVVNFNMATNIESYTHRIGRTGRAGKSGVAITFLGPEDNDVL~YDLRQIISKSSISK
FG06849.1 	DVSLVVNFNMATNIESYTHRIGRTGRAGKSGVAITFLGPEDHETM~YDLKQILSKSSISK
AN1634.1  	DVSLVINFNMATSIESYTHRIGRTGRAGKSGVAITFLGNEDADVM2YDLKQMLIKSPISR
          	*****:***** .** *************.*:**** * **  .: *.*:*:: ** :*:

MG01583.1 	VPPWLKDHPEAQSKPTF--------------------
NCU02803.1	VPDELRRHEAAQNKPQKGQ-KKLEESNGYSGKGGSWN
FG06849.1 	VPEELRRHEAAQSKPVRGAKKDKDEGS---GKGNWQH
AN1634.1  	VPEELRKHEAAQSKPTRGAGKKIEEASGFAGKGGW--
          	**  *: *  **.**  .  .. ..... :....
```
